# Real-world comparison of conduction system pacing and helix-based leadless pacemakers in single-chamber pacing: Procedural outcomes from the PACEMAN study

**DOI:** 10.1016/j.hroo.2026.03.010

**Published:** 2026-03-19

**Authors:** Behnam Subin, Corinne Isenegger, Felix Mahfoud, Sven Knecht, Beat Schär, David Spreen, Jonas Gruthoelter, Jana Wälty, Philipp Krisai, Anna Traub, Julius Nikorowitsch, Nicolas Schaerli, Christian Sticherling, Roland Tilz, Michael Kühne, Patrick Badertscher

**Affiliations:** 1 Department of Cardiology, University Hospital Basel, Basel, Switzerland; 2 Cardiovascular Research Institute Basel, University Hospital Basel, Basel, Switzerland; 3 Department of Rhythmology, University Hospital Schleswig-Holstein, Lübeck, Germany; 4 German Center for Cardiovascular Research, Partner Site Hamburg/Kiel/Lübeck, Lübeck, Germany

**Keywords:** Pacemaker, Leadless pacemaker, Conduction system pacing, Cardiac implantable electronic devices, Electrophysiology

**Key Findings**

▪ In this multicenter real-world cohort of 103 patients with preserved left ventricular ejection fraction undergoing single-chamber ventricular pacing, leadless pacemaker (LPM) implantation was associated with shorter total procedure duration than conduction system pacing (CSP), but longer fluoroscopy time.
▪ Acute procedural success was 100% for LPM implantation, whereas confirmed conduction system capture was achieved in 71% of CSP cases, with failures mainly related to the inability to obtain stable capture with acceptable thresholds.
▪ Acute complication rates were low and comparable between strategies, with 1 LPM dislodgement requiring retrieval and reimplantation and, in the CSP group, 1 pocket hematoma and 1 lead dislodgement.

In patients requiring single-chamber ventricular pacing with preserved left ventricular ejection fraction (LVEF), conduction system pacing (CSP) and leadless pacemakers (LPMs) represent contemporary alternatives.^1^^,^^2^

We conducted a multicenter observational cohort study including patients undergoing single-chamber ventricular pacing at the University Hospital Basel and the University Hospital Schleswig-Holstein, Lübeck, between April 2022 and March 2025. Device selection (CSP or LPM) was at the operator’s discretion. 6 experienced electrophysiologists performed all procedures. Baseline characteristics, procedural parameters, and pre- and postimplant 12-lead electrocardiograms were systematically collected. All patients provided a written informed consent, and the study was approved by the local ethics committees in accordance with the Declaration of Helsinki.

Procedural characteristics, electrical performance, and acute complications of CSP vs LPM implantation are presented in Figure 1. Procedure time was defined as skin incision or vascular puncture to final wound closure or sheath removal. Preparation time was defined as laboratory entry to skin incision, including patient positioning, sterile preparation, and system setup.

**Figure 1:**
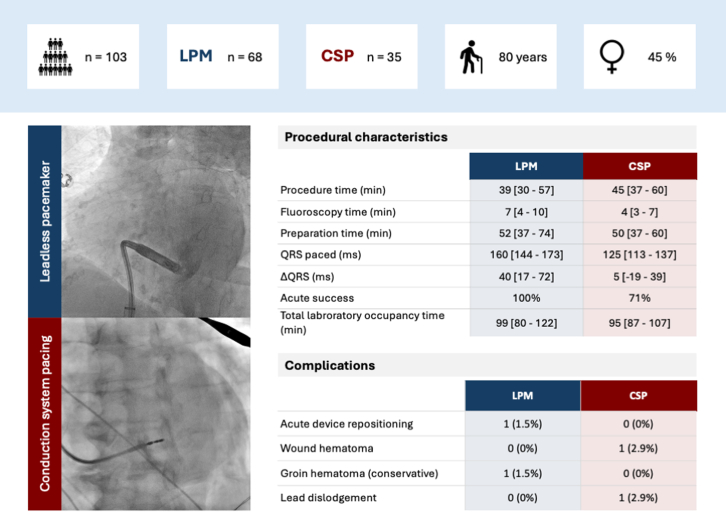
Comparison of CSP and LPM implantation in patients with single-chamber ventricular pacing indications. CSP = conduction system pacing; LPM = leadless pacemaker.

A total of 103 patients were included, of whom 68 (66%) underwent LPM implantation and 35 (34%) CSP. Baseline characteristics were largely comparable; however, pacing indication and New York Heart Association functional class differed significantly. Pacing indication differed significantly between groups (*P* = .003), with a higher proportion of pace-and-ablate procedures in the CSP group, whereas sick sinus syndrome and bradycardic atrial fibrillation were more frequent in the LPM group. Median LVEF was preserved and similar between groups (55% [interquartile range 47–60] vs 55% [50–60]; *P* = .158).

Acute procedural success was 100% for LPM implantation and 71% for CSP with confirmed conduction system capture. Acute CSP failure resulted from the inability to achieve stable conduction system capture with acceptable pacing thresholds, leading to conversion to deep septal or conventional right ventricular pacing. LPM implantation was associated with a shorter total procedure duration (39 vs 45 minutes; *P* = .048) but longer fluoroscopy time (7 vs 4 minutes; *P* < .001). Preparation time and total laboratory occupancy were similar between groups. Electrical performance differed significantly: CSP resulted in narrower paced QRS durations (125 vs 160 ms; *P* < .001) and smaller ΔQRS values than baseline (5 vs 40 ms; *P* = .009), consistent with more physiological ventricular activation. Acute complication rates were low and did not differ significantly between groups (1.5% vs 5.7%; *P* = .603). 1 LPM dislodgement required snare retrieval and immediate reimplantation. In the CSP group, 1 pocket hematoma and 1 lead dislodgement occurred. No in-hospital deaths were observed.

Learning-curve analysis showed procedural times for CSP stabilized after approximately 15–20 cases, whereas LPM implantation times remained consistent throughout the study period, reflecting a standardized workflow. These findings should be interpreted cautiously, given that procedures were performed by multiple operators across centers, and differences in operator experience and device heterogeneity may have influenced procedural efficiency. This study was designed to evaluate procedural characteristics and in-hospital outcomes and was not powered to assess long-term clinical endpoints such as heart failure hospitalization, device revision, or pacing-related cardiomyopathy.

This multicenter analysis represents the first direct comparison of CSP and an active-fixation helix-based LPM in patients with preserved LVEF undergoing single-chamber pacing. LPM implantation was associated with greater procedural efficiency, whereas CSP achieved superior electrical parameters indicative of more physiological ventricular activation. Acute safety profiles were favorable and comparable for both strategies.

The shorter procedure duration observed with LPM implantation likely reflects a simplified implantation approach without device pockets or transvenous leads. CSP, although technically more demanding, may be advantageous in patients with high anticipated pacing burden, given the association between wider paced QRS duration and pacing-induced cardiomyopathy.^3^ Beyond efficiency, LPMs avoid lead- and pocket-related complications, lower infection risk, preserve venous anatomy, and can streamline pace-and-ablate strategies.

Key limitations include the observational design, modest sample size, lack of long-term follow-up, baseline differences in pacing indication and functional status, and potential operator-related confounding. Leadless pacing was predominantly performed using a helix-based active-fixation system; procedural characteristics may differ across leadless platforms, limiting generalizability. Therefore, learning-curve analyses should be considered exploratory. Future studies, including next-generation leadless systems capable of conduction system capture, are needed to clarify long-term clinical outcomes.

## References

[1] Reddy V.Y., Exner D.V., Cantillon D.J. (2015). Percutaneous implantation of an entirely intracardiac leadless pacemaker. N Engl J Med.

[2] Reddy V.Y., Exner D.V., Doshi R. (2022). Primary results on safety and efficacy from the LEADLESS II–phase 2 worldwide clinical trial. JACC Clin Electrophysiol.

[3] Kiehl E.L., Makki T., Kumar R. (2016). Incidence and predictors of right ventricular pacing-induced cardiomyopathy in patients with complete atrioventricular block and preserved left ventricular systolic function. Heart Rhythm.

